# The structures of the SNM1A and SNM1B/Apollo nuclease domains reveal a potential basis for their distinct DNA processing activities

**DOI:** 10.1093/nar/gkv1256

**Published:** 2015-11-17

**Authors:** Charles K. Allerston, Sook Y. Lee, Joseph A. Newman, Christopher J. Schofield, Peter J. McHugh, Opher Gileadi

**Affiliations:** 1 Structural Genomics Consortium, Old Road Campus Research Building, Roosevelt Drive, University of Oxford, Oxford OX3 7DQ, UK; 2 Chemistry Research Laboratory, University of Oxford, Mansfield Road, Oxford, OX1 3TA, UK; 3 Department of Oncology, Weatherall Institute of Molecular Medicine, University of Oxford, John Radcliffe Hospital, Oxford OX3 9DS, UK

## Abstract

The human SNM1A and SNM1B/Apollo proteins are members of an extended family of eukaryotic nuclease containing a motif related to the prokaryotic metallo-β-lactamase (MBL) fold. SNM1A is a key exonuclease during replication-dependent and transcription-coupled interstrand crosslink repair, while SNM1B/Apollo is required for maintaining telomeric overhangs. Here, we report the crystal structures of SNM1A and SNM1B at 2.16 Å. While both proteins contain a typical MBL-β-CASP domain, a region of positive charge surrounds the active site of SNM1A, which is absent in SNM1B and explains the greater apparent processivity of SNM1A. The structures of both proteins also reveal a putative, wide DNA-binding groove. Extensive mutagenesis of this groove, coupled with detailed biochemical analysis, identified residues that did not impact on SNM1A catalytic activity, but drastically reduced its processivity. Moreover, we identified a key role for this groove for efficient digestion past DNA interstrand crosslinks, facilitating the key DNA repair reaction catalysed by SNM1A. Together, the architecture and dimensions of this groove, coupled to the surrounding region of high positive charge, explain the remarkable ability of SNM1A to accommodate and efficiently digest highly distorted DNA substrates, such as those containing DNA lesions.

## INTRODUCTION

**Figure 1.**
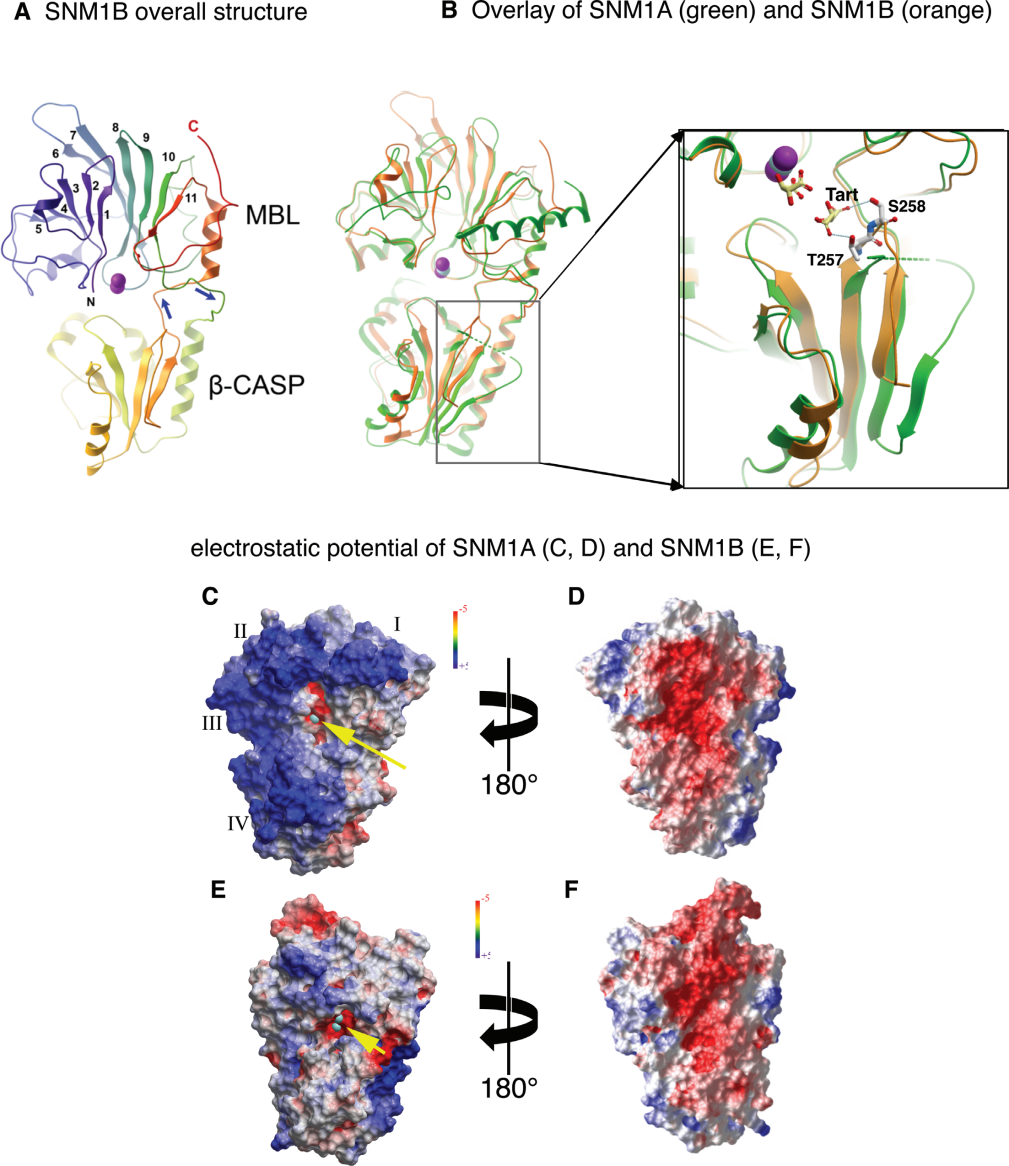
Overall structural features of SNM1A and SNM1B. (**A**) SNM1B, shown in cartoon representation. The N- and C-termini are indicated with N and C and in blue to red coloring. The numbers indicate the consecutive β strands of the MBL domain; the two zinc ions are shown as purple spheres. The β-CASP domain (bottom) is an insert between MBL strands 10 and 11, indicated by the blue arrows. (**B**) Overlay of SNM1A (green) and SNM1B (orange). The crystal structure of SNM1A includes only one zinc ion, shown in cyan. A region in the CASP domain (boxed, magnified) where the main chain trajectory is different between SNM1A and SNM1B. Hydrogen bonds between residues T257, S258 and a tartrate ion are shown. (**C–F**) electrostatic surface potential of SNM1A (**C**, **D**) and SNM1B (**E**, **F**), shown from two opposing orientations. Annotation in (C): yellow arrow points at the active site zinc shown in cyan). I, II, III and IV indicate patches of positive potential on the surface of SNM1A; the basic residues contributing to each patch are described in the text.

DNA exonucleases with a 5′-3′ polarity play a key role in the maintenance of genome stability. Prominent roles include resecting DNA double-strand break ends prior to their repair and in processing intermediates generated during mismatch repair (1,2). The major 5′-3′ DNA repair exonucleases found in humans fall into three families; the Rad2/XPG family (Exo1, FEN1), DNA2 family, the VRR_nuc family (FAN1) and those containing a metallo-β-lactamase (MBL) fold (SNM1A, SNM1B and SNM1C, also known as DCLRE1A, DCLRE1B and DCLRE1C, respectively) (3). The latter family is by far the least characterized, despite its members playing critical roles in DNA repair, immune system development and telomere maintenance (4–6). Although the MBL-fold in these repair proteins is sequence-related to the ‘true’ MBL-folds found in prokaryotic enzymes that hydrolyze and detoxify β-lactam antibiotics (penicillins, for example), non-canonical MBL folds are also present throughout evolution in enzymes acting on a broad range of substrates (6). In addition to DNA processing, there are MBL-fold enzymes that act on tRNA and mRNA maturation (e.g. human ELAC2 and CPSF73) (7–9). Eukaryotic nucleic acid processing MBL-fold proteins are distinguished from the other members of the MBL-fold superfamily by the presence of a second highly-conserved domain distal to the MBL-fold, known as the β-CASP region after its founder members (CPSF-Artemis-Pso2-SNM1) (Figure 1A) (5,6,8). The MBL-fold contains four motifs, where motif 2 (HxHxDH) has been suggested to co-ordinate metal ions important for catalysis (5,8,10). Consistently, mutation of these residues abolishes the nuclease activity of all MBL-fold nucleases *in vitro* (8,10–12). The two human DNA exonucleases containing an MBL-fold are SNM1A (also called DCLRE1A), a factor important for DNA interstrand cross-link (ICL) repair (13,14), and SNM1B (also called Apollo or DCLRE1B), a DNA repair factor that also plays an key role in maintaining overhangs at telomeres (15–18). A third member, SNM1C (also called Artemis), is endowed with a weak DNA exonuclease activity, and also acts as an endonuclease in the presence of DNA-PKcs, cleaving hairpin-overhang intermediates generated during V(D)J recombination, and Artemis defects can lead to a form of Severe Combined Immune Deficiency associated with radiosensitivity (RS-SCID) (11,19,20).

SNM1A has been proposed to act following the initial endonucleolytic incision of ICLs (for example, by XPF-ERCC1 in the case of replication-coupled ICL repair), digesting the incised strand past the point of the ICL to leave a single tethered nucleotide (10,14). This remnant is known to be a good substrate for the translesion polymerases that perform the subsequent step of ICL repair. Very recently, it has been shown that SNM1A directly binds to the Cockayne Syndrome B protein (CSB), which stimulates the exonuclease activity of SNM1A, to facilitate transcription-coupled ICL repair (21). By contrast, the role of SNM1B in DNA repair is less clear. SNM1B-depletion has been shown to induce sensitivity to both ionizing radiation and crosslinking agents (17), and SNM1B has been proposed to interact with a number of genome stability factors including SLX4, Mus81, Mre11 and FANCD2, although it is unclear whether these interactions are direct (22–24). Better understood is the role of SNM1B in telomere maintenance. Here, SNM1B is targeted to telomere ends through its direct interaction with TRF2, and abolishing this interaction induces the hallmarks of telomere dysfunction (15,16,18,25). SNM1B appears to prevent the inappropriate activation of DNA damage repair response by helping generate the 3′ overhang at leading-end telomeres, facilitating T-loop formation and protecting against end-joining (26–29). Disruption of the SNM1B-TRF2 interaction gives rise to Hoyeraal-Hreidarsson (HH) syndrome, associated with premature aging, bone marrow failure, and immunodeficiency (27).

The currently established activity of the MBL-fold DNA exonucleases (SNM1A and SNM1B) as 5′-3′ exonucleases is similar, with both exhibiting a preference for single-stranded DNA *in vitro*, and little capacity to digest single- or double stranded RNA (10). However, several differences are apparent. First, the capacity of SNM1A to process DNA containing site-specific ICLs is much greater than that of SNM1B (10). Moreover, SNM1A demonstrates markedly increased processivity (or activity) on high molecular weight DNA, such as on plasmid substrates, an effect not observed for SNM1B (10).

Here, we present the crystal structures of SNM1A and SNM1B and used this information to define important elements for their activity and to shed light on their mechanism. An extended active site in both enzymes includes, in addition to the canonical di-zinc catalytic centre, a binding pocket that defines the selectivity for terminal 5′ phosphate, and a wide binding groove which is essential for processivity and for the ability to digest past DNA damage sites. Different charge distributions along the DNA binding groove may account for the drastic difference in processivity and DNA digestion efficiency, including that of damaged substrates, between SNM1A and SNM1B.

## MATERIALS AND METHODS

### Cloning and expression of SNM1A and SNM1B

The core MBL-β-CASP domains of SNM1A (aa 676–1040) and SNM1B (aa 1–335) were cloned into the baculovirus transfer vector pFB-LIC-Bse (30). The SNM1B clone included a PCR-derived mutation S330F, a residue that is in an unstructured region of the crystal structure. Baculoviruses were generated by recombination in *E. coli* DH10Bac (Life Technologies) followed by transfection and two rounds of amplification in SF9 cells. The vectors encode an N-terminal tag that includes a His_6_ sequence and a TEV protease cleavage site, which leaves the sequence SM preceding the native protein sequence. The proteins were expressed in 1-l cultures of SF9 cells in 4-l shaker flasks at 27°C, infected at 2 × 10^6^ cells/ml with 3 ml of virus, and incubated for further 70 h. The cells were collected by centrifugation, suspended in 15 ml/l of lysis buffer (50 mM HEPES, pH 7.5, 0.5 M NaCl, 5% v/v glycerol, 10 mM imidazole, and 1 mM TCEP) and frozen at −80°C.

### Protein purification

Cells were thawed, 3–5 volumes of lysis buffer were added, and the cells were disrupted by sonication. The lysate was centrifuged for 30 min at 40 000 × g, and the clear supernatant was collected. The clarified cell lysate was loaded on a 5-ml NiNTA column by gravity flow. The column was washed with 20 volumes of wash buffer (lysis buffer with 30 mM imidazole), and the protein was recovered with elution buffer (lysis buffer with 300 mM imidazole). The eluted protein was combined with His10-tagged TEV protease (1/10 w/w) in a dialysis tubing, and digestion of the tag was performed overnight at 6°C while dialysing against 4 l of wash buffer. The material was then passed through a 1-ml HisTrap column to remove contaminating proteins. The column was developed with a 20-ml gradient from wash buffer to elution buffer, and all fractions were analysed by SDS-PAGE. SNM1A bound to the Ni column after removal of the tag, and was eluted at approximately 80 mM imidazole; SNM1B bound less tightly, and was eluted in early fractions.

The SNM-containing fractions from the second IMAC column were combined, concentrated to <4 ml using a centrifugal concentrator (MWCO = 10 kDa), and loaded on a Superdex S200 HR 16/60 column equilibrated with GF buffer (10 mM HEPES, pH 7.5, 300 mM NaCl, 0.5 mM Tris(2-carboxyethyl)phosphine (TCEP), 5% glycerol) at 1.2 ml/min. Both proteins eluted as monomers. The proteins were concentrated to the desired concentration (10 mg/ml), then divided in small aliquots (<100 μl) in thin-walled PCR tubes and flash-frozen in liquid N_2_. Purification yields were 2–4 mg/l for both proteins.

Intact mass analysis of SNM1B by electrospray-ionization time of flight yielded the expected molecular mass of 37848 (including the inadvertent S330F mutation).

Intact mass analysis of SNM1A^676–1040^ revealed a major peak of the expected mass of 41324.2, which matches precisely the expected mass of 41324. A second peak at the lower mass of 39444 was also observed. After crystallization, the crystals were re-analysed by mass spectrometry, and the protein was found to be converted entirely to a 39444 Da form. This can be explained by a loss of the 20 N-terminal amino acids, with the protein starting at S696; this is in agreement with the crystal structure. Subsequent experiments using a construct initiating at S696 yielded, with high efficiency, the same crystal form. To eliminate an interaction of the N-terminal with the zinc ion of a neighbouring protein molecule, we further truncated the protein. A construct encompassing aa 698–1040 crystallized in a different crystal form with different crystal contacts in which the zinc region is solvent-exposed.

### Protein crystallization

SNM1A: crystallization was achieved by vapour diffusion in a sitting drop arrangement, by mixing 100 nl of protein (9.4 mg/ml) with 50 nl of reservoir solutions containing 18–27% PEG 3350, 0.1 M bis–tris propane pH 7.5, 0.15–0.25 M NaI, and 5–10% ethylene glycol. Crystals were soaked in a cryo-protectant solution containing 25% ethylene glycol in the reservoir solution and plunged into liquid nitrogen. For initial phasing, crystals were soaked for 1 min with 2.5 mM potassium tetrachloroplatinate in cryoprotectant solution.

A second crystal form of SNM1A was obtained after further truncation of the N-terminus, eliminating a crystal contact in which S696 contributed two coordination bonds to the zinc in a neighbouring molecule. This protein, spanning, crystallized in conditions including 0.20 M NaF; 0.1 M bis–tris propane, pH 8.5; 20.0% PEG 3350; 10.0% ethylene glycol.

SNM1B: crystallization was achieved by vapor diffusion in a sitting drop arrangement, by mixing 150 nl of protein (10.0 mg/ml) with 75 nl of reservoir solutions containing 0.4M K/Na-tartrate at 4°C. Crystals were soaked in a cryo-protectant solution containing 25% ethylene glycol in the reservoir solution and plunged into liquid nitrogen.

### Data collection and refinement

SNM1A: Data for experimental phasing was collected from a platinum-soaked crystal to 2.5Å; subsequent native datasets were collected to a resolution of 2.16 Å in beamline I02 in Diamond Light Source. The data was integrated with MOSFLM, merged and scaled with SCALA. An initial solution was obtained by experimental phasing (SAD), which allowed a clear building of the chain and location of the zinc. This was used as a model for molecular replacement (PHASER) and subsequent refinement (REFMAC) of the native dataset. Electron density is seen for residues 696 – 1040, with an intermediate peptide (964–973) not visible in the structure, and assumed to be disordered. The construct expressed a protein spanning aa 675–1040, but (as described above), we found that the N-terminal up to G695 was degraded during incubation, by an endogenous protease. 100% and 97% of residues are in allowed and favoured areas, respectively, of the Ramachandran plots.

Crystal form B of SNM1A diffracted to 2.19 Å in beamline I04-1 of Diamond Light Source. Data was integrated and scaled with XDS, and the structure was solved and refined by molecular replacement using PHENIX.

SNM1B: Data to a resolution of 2.16 Å were collected at Diamond Light Source, beamline I24. Data were integrated and scaled with XDS and SCALA. The structure was solved by molecular replacement (PHASER) using SNM1A (4B87) as a model. Electron density is seen for residues 1–313; the C-terminal peptide (314–335) is not visible in the structure and is assumed to be disordered. 100% and 98% of residues are in allowed and favoured areas, respectively, of the Ramachandran plots.

All structure figures were generated using ICM-pro software (Molsoft LLC, San Diego). Surface electrostatic potential was calculated and displayed using the REBEL (Rapid Exact-Boundary Electrostatics) algorithm in ICM-pro (31).

### Substrate preparation

All substrate sequences are listed in Supplementary Table S1 and were synthesised by Eurofins MWG Operon (Ebersberg, Germany). Double-stranded DNA (dsDNA) substrates were annealed by heating to 100°C for 5 min, followed by step-wise cooling to room temperature (20°C). DNA containing SJG-136 ICLs was prepared according to published methods (14,32).

Single-stranded DNA (ssDNA) substrates were labelled at the 3′ end with [^32^P]-α-dATP (1 μl, 0.71 MBq) and terminal deoxynucleotidyl transferase (TdT) (20U, Fermentas). dsDNA substrates were annealed and labelled at the 3′ end with [^32^P]-α-dATP (1 μl, 0.71 MBq) and Klenow fragment without 3′ exonuclease activity (5U, New England Biolabs).

### Nuclease assays

The nuclease assays used in this paper are based on those previously described (10), with the relevant oligonucleotides listed in Supplementary Table S1. Briefly, exonuclease activity was measured using SNM1A(676–1040) (0.35 ng, 0.8 nM) or SNM1B(1–335) (0.15 ng, 0.4 nM) mixed with 1 pmol (100 nM) of 3′-[^32^P]-labeled DNA substrate in 10 μl of 20 mM HEPES pH 7.9, 50 mM KCl, 10 mM MgCl_2_, 0.5 mM DTT, 0.05% Triton-X, 0.1 mg/ml BSA and 5% glycerol. Reactions were incubated at 37°C for the indicated times and stopped by adding 2 μl of 80% formamide/10 mM EDTA to each reaction and heating at 95°C for 5 min. Following separation on a 20% polyacrylamide/7M urea denaturing gel, substrate and product bands were visualized by a Typoon Trio+ Variable Model Imager.

Nuclease assays with the modified pUC18 plasmids were carried out in two steps. First, the supercoiled plasmid (325 ng, 9 nM) was either left untreated, nicked with Nb.BbvCI (5U, New England Biolabs) or linearised with HindIII (10U, New England Biolabs) in 10 μl at 37°C for 2 h. Reaction buffer (20 mM HEPES pH 7.9, 50 mM KCl, 10 mM MgCl_2_, 0.5 mM DTT, 0.05% Triton-X100, 0.1 mg/ml BSA and 5% glycerol) and SNM1A(676–1040) (0.5 ng, 12 nM) were then added to each type of plasmid and left to digest for 1 h at 37°C. The reactions were quenched with 60 mM EDTA/50% glycerol (6× loading buffer). The samples were separated on 1% agarose gel containing 1× TBE.

### Electrophoretic mobility shift assay (EMSA)

The indicated concentration of SNM1A(676–1040) or SNM1B(1–335) was incubated with 10 fmol (1 nM) of 3′-[^32^P]-labeled 51-mer single-stranded DNA substrate in 20 mM HEPES pH 7.9, 50 mM KCl, 0.5 mM DTT, 0.05% Triton-X, 0.1 mg/ml BSA and 5% glycerol for 5 min at 37°C before quenching on ice. 2 μl of loading dye (50% glycerol and 10 mM EDTA) was added to each sample. The samples were separated on 8% polyacrylamide non-denaturing gel at 100 V for 3 h. The gels were dried and, bands were visualized by a Typhoon Trio+ Variable Model Imager.

### Real-time kinetic measurements

Real-time kinetic measurements were performed as described (10) using the 20-mer oligonucleotides listed in Supplementary Table S1. Reactions were carried out in black 384-well microplates, and measurements were made using a SpectraMax M2e fluorescent plate reader in fluorescent top read mode, with SoftMaxPro software (Molecular Devices, Sunnyvale, CA) to control the settings. Reactions were performed in 15 μl of the above buffer with varying concentrations of DNA substrate (10, 25, 50, 100, 250, 300, 400, 500, 750, 1000 nM), 0.242 nM SNM1A(676–1040) and its mutants, or SNM1B(1–335) and its mutants. Each reaction was started by the addition of the enzyme, and the fluorescein emission spectra measured (excitation at 495 nm, emission at 525 nm and cutoff at 515 nm) with six readings taken at 7 s intervals for 6 min. The fluorescence intensity of each well was plotted against time, and the rate of increase was determined, plotted against substrate concentration, and fitted to a Michaelis–Menten curve on Prism software (GraphPad Software, Inc., La Jolla, CA) to determine *K*_M_ and *k*_cat_.

## RESULTS

### The structures of SNM1A and SNM1B reveal a typical MBL fold but major differences in charge distribution

The catalytic domains of human SNM1A (amino acids 676–1040) and SNM1B (aa 1–335) proteins were purified from insect cells and crystallized (Table 1). A 2.16 Å crystal structure of SNM1B contains a single protein molecule in the asymmetric unit, with two coordinated zinc ions and two tartrate molecules at the active site. Structures of SNM1A were solved in two different crystal forms at resolutions of 2.16 and 2.19 Å; the structures are virtually identical, except that crystal form B is lacking two N-terminal amino acids present in form A (RMS difference of 0.57 Å; see Table 1 and Supplementary Figure S1).

**Table 1.** Data collection and refinement statistics

	4B87; SNM1A, crystal form A	5AHO; SNM1B	5AHR; SNM1A, crystal form B
**Data collection**			
Space group	*P* 61 2 2	*P* 41 3 2	*P* 41 21 2
Cell dimensions			
*a*, *b*, *c* (Å)	83.40 83.40 257.84	143.09 143.09 143.09	113.20 113.20 125.74
*α*, *β*, *γ* (°)	90.0 90.0 120.0	90.0 90.0 90.0	90.0 90.0 90.0
Resolution (Å)	72.23 – 2.16 (2.21-2.16)*	50.59 – 2.16 (2.23-2.16)*	80.04 – 2.19 (2.24-2.19)*
*R*_merge_	0.09	0.22	0.10
*I* / σ*I*	2.01 (at 2.16)	1.88 (at 2.16)	2.24 (at 2.18)
Completeness (%)	100 (72.23 – 2.16)	100.0 (50.59 – 2.16)	100 (80.04-2.19)
Redundancy	11.9 (12.2)*	18.4 (14.8)*	13.3 (13.5)*
**Refinement**			
Resolution (Å)	2.16	2.16	2.19
No. of reflections	29711	27439	42634
*R*_work_/*R*_free_ (in high-resolution shell)	0.177/0.218 (0.29/0.308)^a^	0.206/0.226 (0.310/0.325)^a^	0.179/0.201 (0.283/0.319)^a^
No. of atoms	2819	2758	2844
Protein	2626	2479	2649
Ligand/ion	17	46	5
Water	176	233	190
*B*-factors	47.4	33	47.0
r.m.s. deviations			
Bond lengths (Å)	0.019	0.002	0.008
Bond angles (°)	1.95	0.592	1.067

^a^Values in parentheses are for highest-resolution shell.

Figure 1A shows the overall fold of SNM1B. The protein exhibits two structural domains: the metallo-β-lactamase (MBL) domain, arranged as a sandwich of two mixed β sheets (α/β-β/α); and the β-CASP domain, which is characteristic for a subset of MBL nucleases: CPSF-73, Artemis, SNM1A, Pso2, as well as RNaseJ (6). Although often annotated as separate domains in the protein sequence, the CASP domain occurs as an insert between strands 10 and 11 of the MBL domain. This structural arrangement is similar to other MBL-β-CASP proteins, including SNM1A (Figure 1B) and the ribonucleases CPSF-73 (Supplementary Figure S2; a structure-based sequence alignment of the SNM1A, SNM1B and CPSF73 is provided in Supplementary Figure S3) and RNase J (8,33,34). Although SNM1A and SNM1B have very similar folds (2.0 Å RMSD), they differ markedly in the surface charge distribution (Figure 1C-F). When looking from the direction of the active site, SNM1A is predominantly positive, while SNM1B is only moderately so. While it is difficult to assign the overall surface potential to specific amino acid side chains, we note the differences between the corresponding residues K705/L5, K706/I6, K743/S42 and K906/E209 of SNM1A/SNM1B, respectively. Remarkably, the reverse side of both proteins is predominantly negative. The electropositive surface regions in SNM1A, identified with Roman numerals in Figure 1C, can probably be attributed to the following residues and structural features: (I) residues K1022, R1024, K1029, R1032 and the dipole of the last α-helix of SNM1A, which is non-helical in SNM1B; (II) residues R697, K698 and K699; (III) residues K743, H744 and K760; and (IV) K940, K947, K948 in the β-CASP domain. Another difference is the loop between the final 2 B-strands in the β-CASP domain, which is quite different between the two (Figure 1B, enlarged box). In SNM1B it is well ordered and makes hydrogen bonds (via T257 and S258) to the second tartrate molecule. This segment of the chain is partly disordered in the SNM1A crystal structures; it is possible that the ordered conformation is stabilized by, or dependent upon, the presence of the ligand.

### Detailed analysis of the active sites of SNM1A and SNM1B reveals features distinct from RNA processing MBL-fold proteins

**Figure 2.**
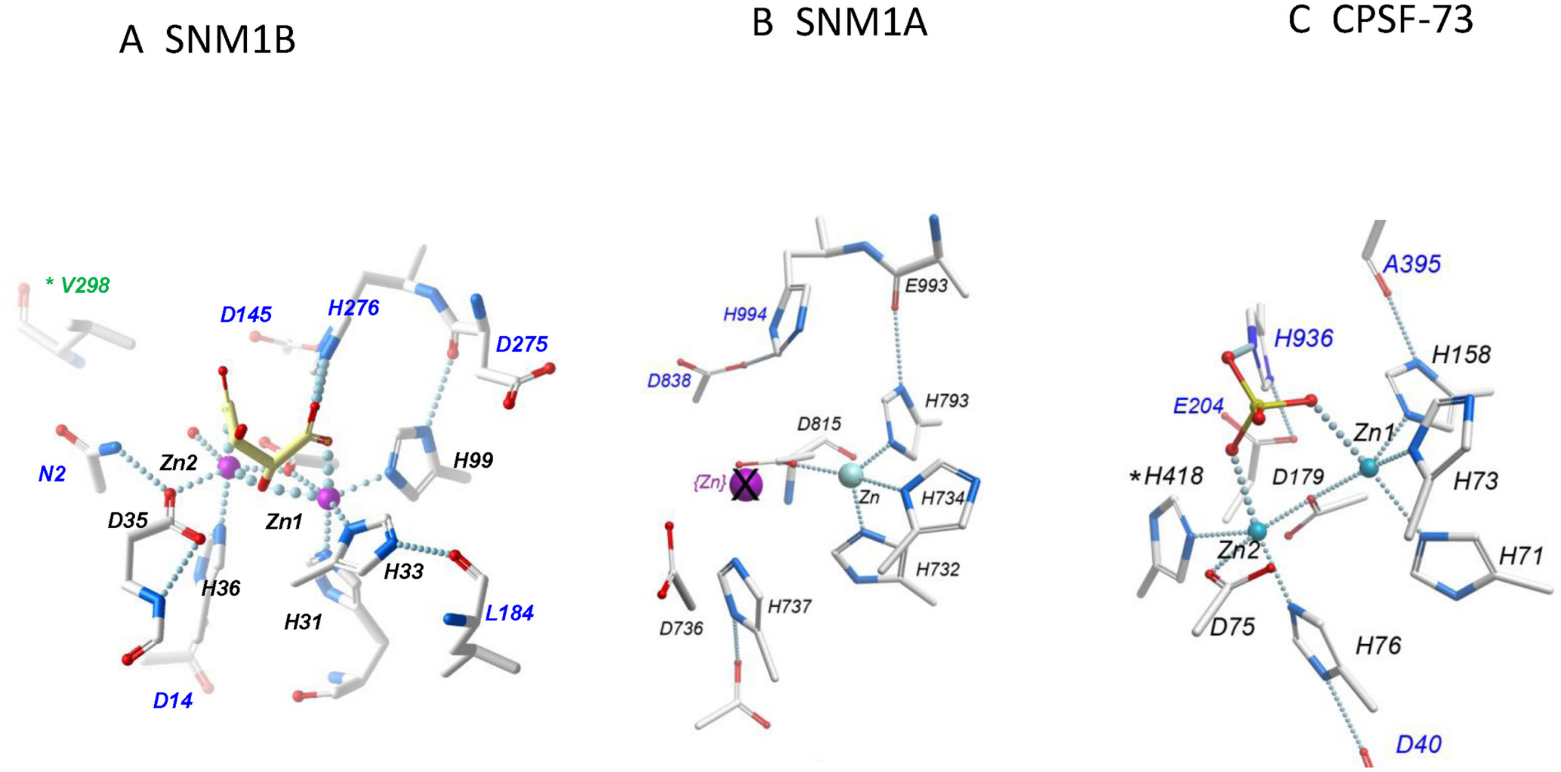
The Zinc-binding regions of SNM1B, SNM1A and CPSF-73. (**A**) SNM1B (PDB: 5AHO): zinc-coordinating residues are labeled in black, other residues involved in the surrounding hydrogen bond network or in binding the tartrate ion are labeled in blue. For clarity, other residues are not shown. (**B**) SNM1A (PDB:4B87): This structure includes only one zinc ion; the purple, crossed-out sphere indicates the position of the second zinc in SNM1B. Note that all the zinc-coordinating residues are conserved with SNM1B, but D736 is rotated relative to the homologous D35 of SNM1B. (**C**) CPSF-73 (PDB: 2I7T). Note that zinc ion here is coordinated by an additional histidine (H418), which is absent in SNM1 proteins.

Figure 2A gives a more detailed look at the region surrounding the two zinc ions in the active site of SNM1B (only residues contacting the zinc and the proximal tartrate ions are shown). MBL proteins contain highly conserved motifs that contribute to the active site: motifs 1–4, A, B and motif 5/C (see Supplementary Figure S3). Motif 2, H31-X-H33-X-D35-H36, contributes two coordination bonds to each of the two zinc ions. H99 (motif 3) contributes the third bond to zinc-1, while a single oxygen of D120 (motif 4) bridges the two zinc ions. H36 and H99 are supported by hydrogen bonds to D14 (motif 1) and the main-chain carbonyl of D275 (Motif B), respectively. Zinc 1 is coordinated, in an octahedral arrangement, to the protein residues H31, H33, D120 and H99; two additional bonds are formed with the carboxyl (O4) and hydroxyl (O3) oxygens of a tartrate molecule (Tart_1) from the crystallization buffer. The shell around the second zinc is also octahedral, coordinated by the D35, H36, the bridging D120, the hydroxyl (O3) and second carboxyl (O1) oxygens of Tart_1, and one water molecule. The binding of tartrate_1 to the zinc centre is suggestive of a possible catalytic mechanism, whereby two oxygens of the phosphodiester to be cleaved are in positions similar to the tartrate carboxyl oxygens (O1 and O4), and a nucleophilic water or hydroxylate ion is coordinated to both zinc ions at the position of the tartrate O3 oxygen. Interestingly, the tartrate is also hydrogen-bonded to H276 (conserved motif B), which is in turn bound to D145 (motif A). If, as discussed below, the zinc-ligated tartrate occupies the position of the phosphodiester cleaved by the enzyme, this provides a rationale for the conserved and essential role of motifs A and B.

All these contacts, and the general geometry of the active site, are precisely conserved in the endonuclease subunit of mRNA cleavage-polyadenylation factor CPSF-73 (Figure 2C) (8). There is one important difference: the second zinc in CPSF-73 is coordinated with an additional residue, H418, defined as motif 5 or C. In SNM1B, this position is occupied by a coordinated water molecule. Histidine is found in the positions aligned with CPSF73-H418 in most subfamilies of MBL-β-CASP enzymes, but is divergent in the DNA repair enzymes SNM1A/B/C and their homologues (Valines 1061 and 298 in SNM1A and SNM1B). Our previous studies demonstrated that substituting Valine 1061 in SNM1A for histidine did not change its substrate preference for DNA versus RNA, or its catalytic efficiency (10) therefore this highly-conserved residue must confer some currently unknown property on this subfamily. It is possible that the SNM-class enzymes bind zinc less tightly, as seems to be the case with SNM1A. It is also possible that the loss of a zinc-coordinating residue confers a more ‘open’ conformation, which allows the SNM1 proteins to accommodate bulkier substrates.

Figure 2B shows the active site environment of SNM1A. Structures of SNM1A in two different crystal forms show only a single zinc ion. Comparison with SNM1B shows that all residues involved in zinc coordination are conserved; all are in the correct positions, except for D736 which is a different side-chain rotamer. As noted above, the absence of a fourth zinc-ligating residue (motif 5) could reduce the binding affinity of zinc in the SNM1 proteins. In SNM1B, D35 is held by a hydrogen bond to N2, which probably stabilizes the zinc-coordinating rotamer; there is no corresponding residue in SNM1A, and the amino-terminal is too distant in SNM1A to form such a contact. However, we expect the catalytic activity of both SNM1A and SNM1B to depend on two metal ions, as mutation of both SNM1A-D736A and SNM1B-D35A completely eliminates activity (10).

### The structures of SNM1A and SNM1B imply how they interact with DNA

**Figure 3.**
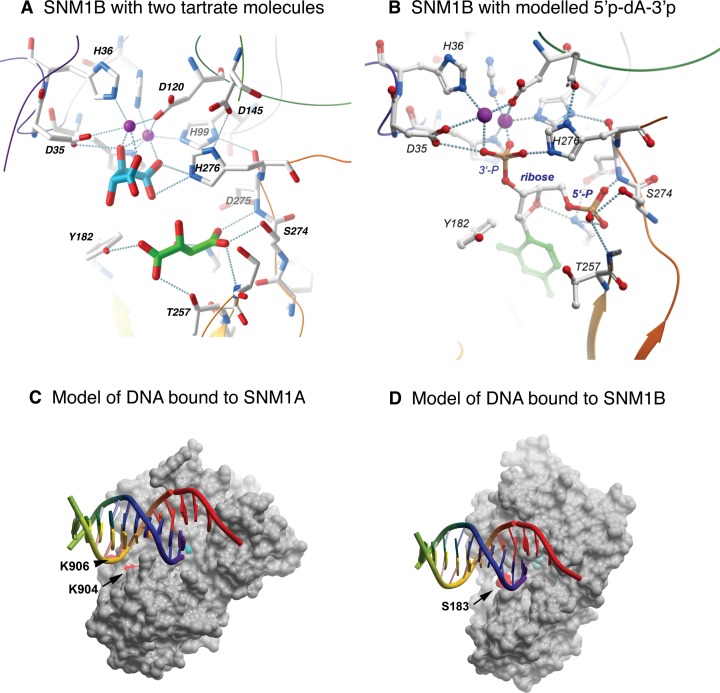
Modeling substrate interactions of SNM1B and SNM1A. (**A**) Expanded view of the active site region of SNM1B, including the sulfate and tartarate ions that are bound in the crystal structure. (**B**) Modeling a 5′, 3′-nucleotide diphosphate based on the positions of the sulfate and tartarate in the crystal structure. The 5′ phosphate could represent the terminal phosphate of a DNA substrate, while the 3′ phosphate may represent the phosphodiester that is hydrolysed by the enzyme. (**C**) Model of DNA bound to SNM1A. The model places the 5′ end of one strand at the active site, while DNA downstream is in position to interact with the distal binding site defined by K904 or K906 (shown as red patches on the protein surface). (**D**) A similar model of DNA bound to SNM1B, where the distal DNA binding site includes S183 (red patch).

Figure 3A shows a more complete view of the active site of SNM1B, now including a second bound tartrate ion. Tartrate_2 is held in place by direct interactions with the side chains of Y182, K186, T257, S258, and S274, and the main chain amines of D275 and S258. Interestingly, K186, and S274 are widely conserved in MBL-β-CASP proteins; Y182 is conserved among DNA repair enzymes but is most frequently replaced by F in RNases (8,33,34), atypically, it is replaced by a D in inactive CPSF100 proteins (8). The tight binding of tartrate may mimic binding of a 5′-terminal phosphate group of the DNA substrate. Indeed, structures of bacterial RNase J proteins bound to mono- or oligonucleotides place a phosphate at a similar position (33,34). Figure 3B presents a model in which a 5′, 3′ nucleotide diphosphate is placed with the two phosphates occupying positions occupied by carboxylate groups of tartrate_2 and tartrate_1, respectively, in the SNM1B crystal structures. This suggests a plausible basis for the selectivity of SNM1B/A for cleavage of the first phosphodiester bond of a DNA molecule bearing a 5′-phosphate. The structural features of the extended active site probably also determine the selectivity for cleavage of DNA over RNA; however, in the absence of a co-crystal structure, there is insufficient information for a detailed model.

The crystal structures of SNM1A and SNM1B both revealed the presence of a groove with the dimensions appropriate for the accommodation of DNA strands, running past the metal-coordinating active site residues (Figure 3C and D, and Supplementary Figure S4). In order to examine whether or not this groove plays a key role in the processing of native and damaged DNA substrates, we employed site-directed mutagenesis to generate a collection of SNM1A and SNM1B proteins that have been mutated for multiple residues along this groove.

### Identification of SNM1A variants with unchanged catalytic activity but reduced processivity and capacity to digest crosslinked DNA

For SNM1A, we generated fourteen variants, singly or multiple mutated for residues adjacent to or lining the putative DNA-interaction groove (Supplementary Figures S5 and 6A). We initially screened these for nucleolytic activity in two assays. First, we utilized a previously described fluorometric assay that allows the quantification of the catalytic properties of the variant enzymes in phosphodiester bond hydrolysis (10). The substrate in this assay contains a fluorescein group at the 5′-terminus of 21-mer ssDNA substrates and a Black Hole Quencher (BHQ) group eight nucleotides 3′-to the fluor moiety (Supplementary Table S1 ‘20-mer fluor substrate’). Digestion of this substrate in the 5′-to-3′ sense uncouples the fluor and quench groups, leading to a fluorescent signal that can be quantified in real time. None of the fourteen mutant forms of SNM1A exhibited a substantially changed Km or Kcat (Supplementary Figure S5), indicating that their ability to catalyse DNA hydrolysis was largely unaffected by these changes. Our second screen used a ssDNA 21-mer oligonucleotide of the same sequence, radio-labelled at its 3′-end. In this analysis, one of the SNM1A variants, SNM1A-K904A-K906T, mutated at two adjacent residues at one end of proposed DNA binding groove exhibited a substantially reduced capacity to fully digest the substrate to completion, showing predominantly premature termination of digestion (Supplementary Figure S6B). Importantly, the data in Supplementary Figures S5 and S6 are not in contradiction, since the fluorescence-based assay used in Supplementary Figure S5 required that only the two 5′-phosphodiester bonds were hydrolysed and therefore this assay could not reveal the differences in processivity seen in Supplementary Figure S6.

**Figure 4.**
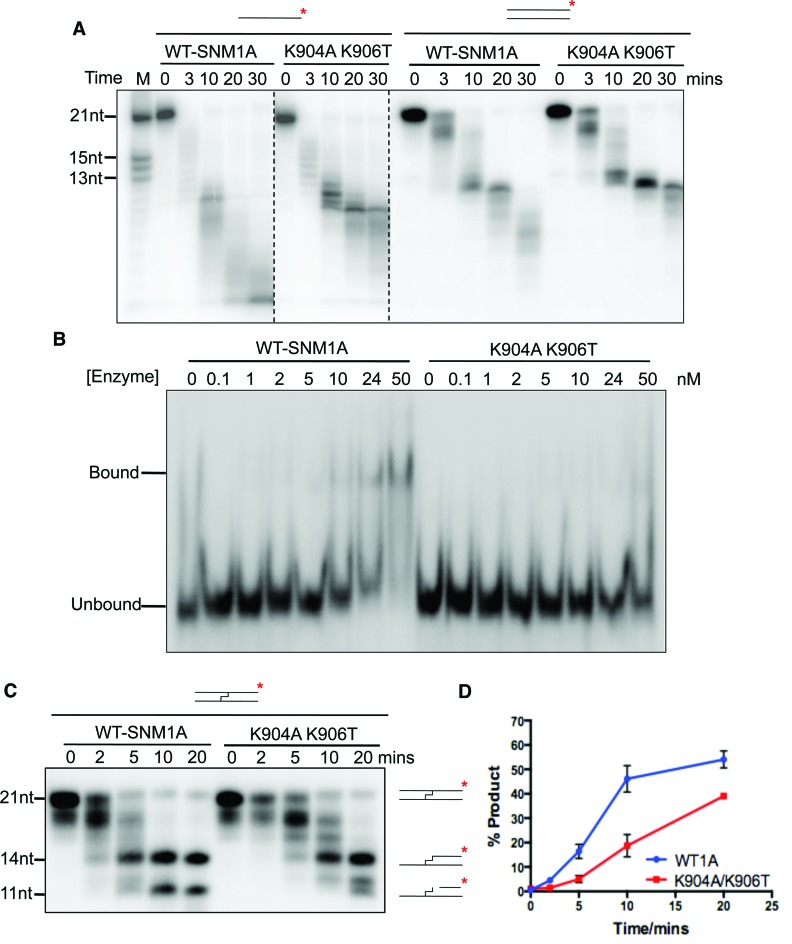
SNM1A-K904A-906T displays reduced nuclease activity. (**A**) Time-course nuclease assay of SNM1A and SNM1A-K094A-K906T (0.8 nM) mutant using 3′ radio-labelled (α-^32^P-dATP) 21-mer single- and double-stranded DNA (100nM) quenched at the indicated times. (**B**) Electrophoretic Mobility Shift Assay (EMSA) with 3′ radio-labeled 51-mer single-stranded DNA with increasing concentrations of SNM1A and SNM1A-K904A-K906T mutant. Enzyme incubated with DNA (1 nM) for 5 minutes at 37°C before quenching on ice. (**C**) Time-course nuclease assay of SNM1A and SNM1A-K094A-K906T (0.8 nM) mutant using 3′ radio-labelled (α-^32^P-dATP) 21-mer double-stranded DNA with a centrally-located SJG-136 cross-link (100 nM), quenched at the indicated times. (**D**) Quantification using ImageJ of the 11nt band from three repeats of nuclease assay of cross-linked DNA with SNM1A and SNM1A-K904A-K906T, error bars show the standard error of the mean.

To explore this observation further, we examined whether the reduced digestion of the short (21-mer) ssDNA oligonucleotides by SNM1A-K904A-K906T was also observed on dsDNA substrates (Figure 4A). This was confirmed, although the difference between the activity of WT and SNM1A-K904A-K906T on dsDNA was less pronounced. We also examined the capacity of SNM1A and SNM1A-K904A-K906T to bind DNA using enzyme mobility shift assays (EMSAs). Consistent with the reduced ability of SNM1A-K904A-K906T to digest oligonucleotide substrates (Figure 4A), the ability of SNM1A-K904A-K906T to bind ssDNA substrates was also reduced (Figure 4B). Therefore, the decreased digestion we observe in SNM1A-K904A-K906T, particularly on undamaged ssDNA, does reflect substantially altered DNA binding characteristics (Figure 4B), but not impaired catalytic efficiency (Supplementary Figure S5).

Since the key biological role of SNM1A is to digest damaged DNA, principally DNA containing ICLs (14), we next examined the capacity of the WT protein and SNM1A-K904A-K906T to digest DNA containing a site-specific SJG-136 ICL (14,32). Using 21-mer oligos of the same sequence as in Figure 4A, but with a centrally located ICL, the pattern of digestion observed with the WT protein was the same as we have previously reported. Here, SNM1A digests up to the point of the ICL, generating a 14-mer intermediate product, but around 50% of the substrate is further digested past the ICL, within 20 min (Figure 4C and D). As previously observed (14), the digestion predominantly terminates with the generation of an 11-mer product, reflecting the probable steric inhibition of further digestion by the attachment site of the SJG-136 ICL to the complementary DNA strand. When we assessed the capacity of SNM1A-K904A-K906T to digest this substrate we observed a reduction in bypass of the ICL, with <20% of the ICL being bypassed within 10 min compared to nearly 50% for the WT enzyme (Figure 4D).

Therefore, while the capacity of SNM1A-K904A-K906T to digest native short dsDNA oligos is slightly compromised, its capacity to undertake a more physiologically relevant reaction, digestion of damaged DNA strand, is more substantially compromised.

**Figure 5.**
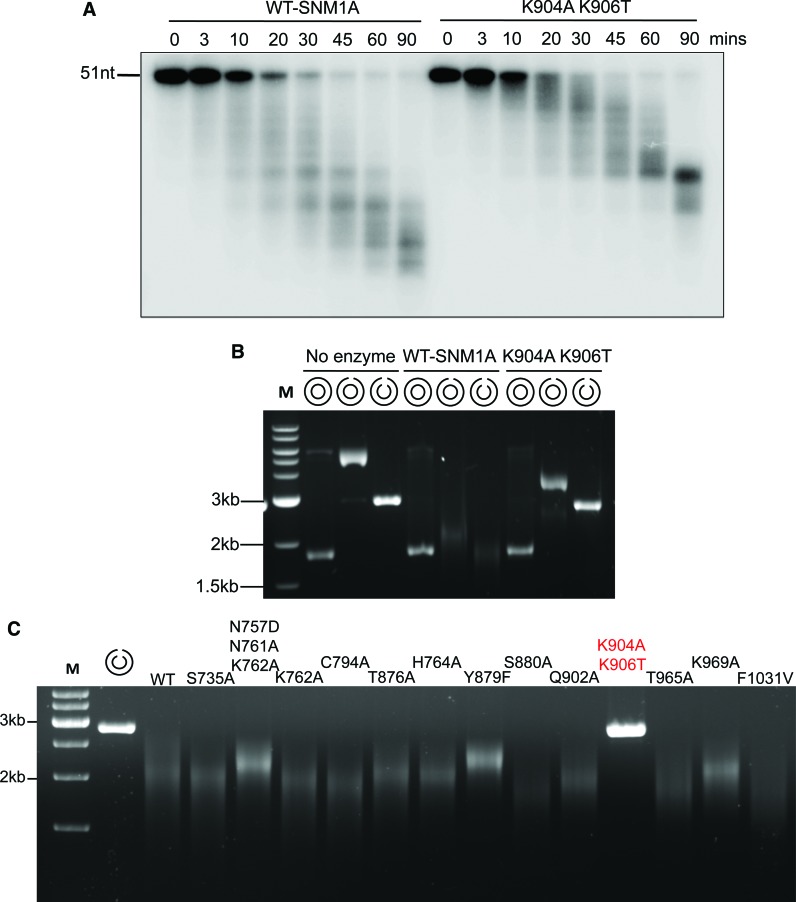
SNM1A-K904A-906T exhibits a severely reduced capacity for digesting high molecular weight DNA. (**A**) Time-course nuclease assay of SNM1A and SNM1A-K094A-K906T (0.8 nM) mutant using 3′ radio-labelled (α-^32^P-dATP) 51-mer single-stranded DNA (100 nM), quenched at the indicated times. (**B**) Nuclease assay of SNM1A and SNM1A-K094A-K906T (12 nM) mutant using supercoiled, nicked and linearised DNA generated from a modified pUC18 plasmid (9 nM). (**C**) Nuclease assay of SNM1A and SNM1A mutants with linearised (using HindIII) modified pUC18 plasmid. Enzyme and DNA in plasmid experiments incubated for 1 hour at 37°C before addition of 6X loading buffer.

Another striking feature of SNM1A activity is its greatly increased processivity on high molecular weight (Mw) DNA substrates, exemplified by plasmid substrates (10). This might be, at least partly, attributable to the DNA-affinity afforded by the large area of positively charged residues surrounding the DNA interacting groove and active site, revealed by the crystal structures (Figure 1C and D). Given that the differences in activity we observe for SNM1A and SNM1A-K904A-K906T are modest on undamaged oligonucleotide DNAs, we hypothesised that the activity of SNM1A-K904A-K906T on a preferred substrate, high MW DNA, might be altered. To this end we, we assessed the activity of SNM1A and SNM1A-K904A-K906T on DNA of increasing molecular weights. Using a 51-mer single-stranded DNA substrate, a similar, modest, decrease in activity was observed as with the 21-mer (Figure 5A). By contrast, the capacity of SNM1A-K904A-K906T to digest a 3 kb plasmid, either from a nick or double-strand break end was drastically curtailed (Figure 5B), indicating that the putative DNA binding groove is required for the processivity of the enzyme on high molecular weight substrates. None of the other variant forms of SNM1A generated demonstrated such drastically altered ability to digest plasmid DNA, although a several exhibited substantially decreased digestion capacity, including SNM1A-N757A-761A-7262A, SNM1A-Y879F and SNM1A-K969A (Figure 5C), further supporting a key role for the integrity of the putative DNA interacting groove in mediating the processivity of SNM1A.

### The DNA putative binding groove of SNM1B is also important for its processivity and digestion of damaged DNA substrates

**Figure 6.**
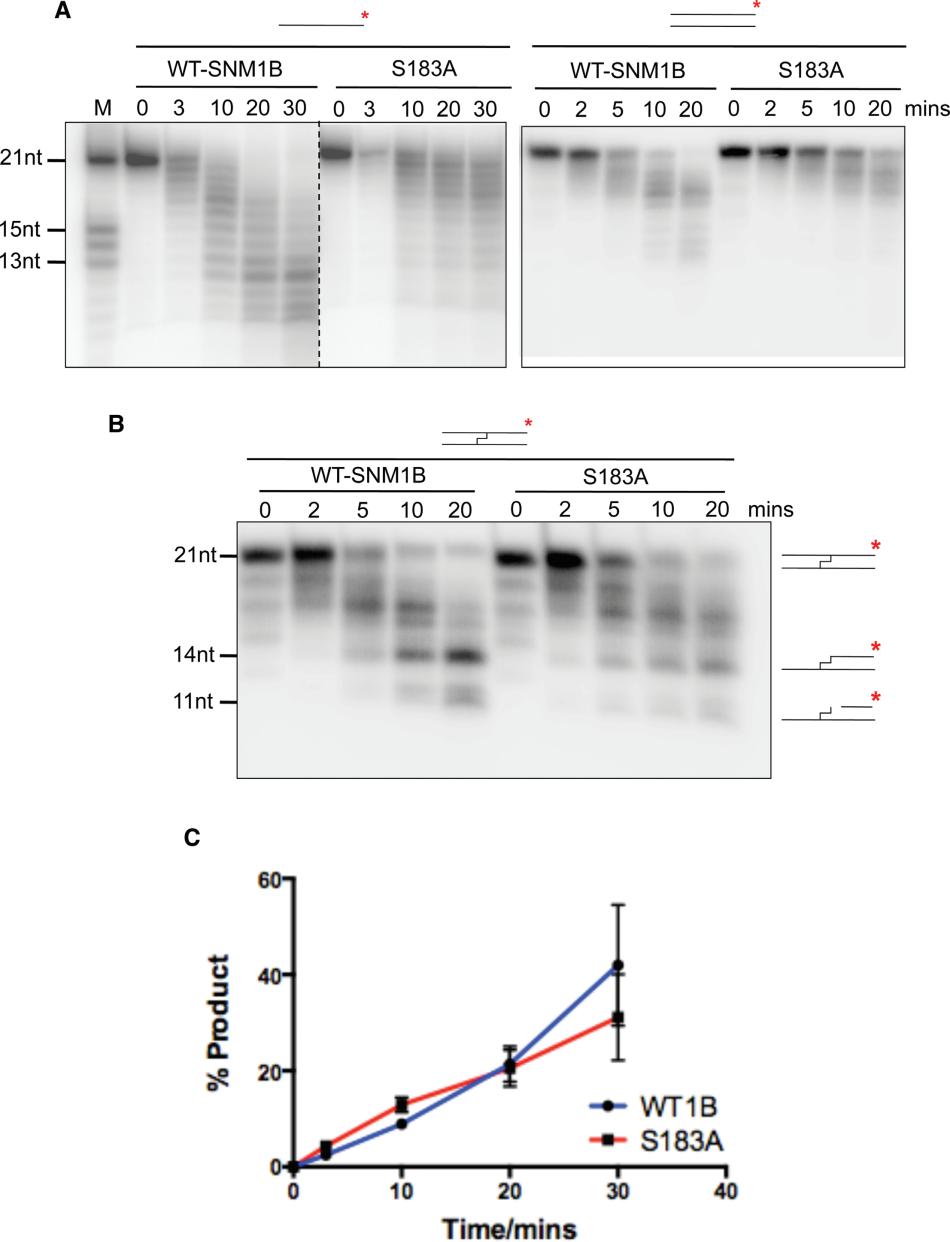
SNM1B-S183A-S330F is impaired for the digestion of both native DNA and ICL-containing substrates. (**A**) Time-course nuclease assay of SNM1B and SNM1B-S183A (0.4 nM) mutant using 3′ radio-labeled (α-^32^P-dATP) 21-mer single- and double-stranded DNA (100 nM) quenched at the indicated times. (**B**) Time-course nuclease assay of SNM1B and SNM1B-S183A (0.4 nM) mutant using 3′ radio-labeled (α-^32^P-dATP) 21-mer double-stranded DNA with a centrally-located SJG-136 cross-link (100 nM), quenched at the indicated times. (**C**) Quantification using ImageJ of the 11nt product band from three repeats of nuclease assay of cross-linked DNA with SNM1B and SNM1B-S183A, error bars show the standard error of the mean.

For SNM1B we undertook a similar analysis, again focusing on the putative DNA binding groove identified in the crystal structure. Here, of the five variant forms of SNM1B generated, one that was mutated for a residues adjacent to this groove, SNM1B-S183A, demonstrated a reduced capacity to digest ssDNA oligos in our gel-based assay (Supplementary Figure S7A and S7B). Interestingly, these residues are in the same region of the binding groove as the SNM1A K904 and K906 residues that most affect processivity and ICL processing. However, as for the SNM1A mutants, the Km and Kcat was not strongly affected by any of the five mutations in our fluoresence-based assay (Supplementary Figure S8). Since, unlike SNM1A, SNM1B does not become processive on high molecular weight DNA, such as plasmid DNA, we could not examine this aspect. More detailed analysis of the activity of WT SNM1B and SNM1B-S183A on undamaged and ICL-containing oligonucleotide DNA substrates revealed that, as for SNM1A, the difference in digestion between the two forms of protein are most pronounced for ssDNA substrates (Figure 6A), strongly preferred substrate of SNM1B (10). Indeed, when we compared the capacity of WT and the SNM1B-S183A variant to digest an ICL-containing substrate, no significant difference was observed (Figure 6B and C); however, we have previously established that, much like native-double stranded DNA, such substrates are poorly processed by SNM1B. This confirms the important role of the putative DNA interacting groove in this family of enzyme in digesting their preferred substrate, ssDNA in the case of SNM1B. We also examined the DNA binding capacity of WT SNM1B and the SNM1B-S183A form. This mutant form demonstrates a reduced capacity to digest and bind 51-mer DNA substrates as determined by EMSA (Supplementary Figure S7C and S7D), and therefore we conclude that the region mutated in this variant which is adjacent to the putative DNA binding groove is indeed important for interactions with DNA.

## DISCUSSION

The SNM1A and SNM1B/Apollo proteins are both classic 5′-3′ exonucleases, requiring a free 5′-phosphate to initiate digestion, but are members of a class of DNA processing factor family that have not previously been structurally characterized. Unlike the third DNA-acting mammalian protein containing a MBL-β-CASP motif, SNM1C/Artemis (11), extensive analysis has not provided evidence for any endonucleolyic activity for SNM1A or SNM1B, at least in isolation. The structures SNM1A and SNM1B revealed proteins that fold as typical MBL-β-CASP proteins, with substantial overall structural similarity to the human RNA processing enzyme CPSF73 (8), for example (Supplementary Figure S2). In particular, the residues forming the signature motifs (1–4, A, B) of this family all play a conserved role in the architecture of the active site (Figure 2). However, one distinguishing feature (common to the DNases) is the replacement of one zinc-coordinating residue, a Histidine defined as motif 5/C, is replaced with a Valine (V1016/V298 in SNM1A/SNM1B). We have previously shown that this residue is not required for DNase activity in SNM1A (10). Hence, the second metal ion may be more loosely associated; in our structures of SNM1A this metal is essentially absent, while SNM1B retains two zinc ions, possibly aided by a stabilizing interaction with N2, which is absent in SNM1A. However, it is likely that the active form of SNM1A also depends on the presence of two zinc ions, as demonstrated by the complete dependence on an aspartate (D736) for nuclease activity that should coordinate the missing zinc (10,14).

The crystal structure of SNM1B includes two tartrate ions in the active site. Tartrate_1 has three oxygens (the two carboxylates and a hydroxyl oxygen) coordinated to the two zinc ions in the active site; it is plausible that, during the catalytic cycle, these are replaced by two oxygens of the phosphate and an activated water / hydroxylate poised for nucleophilic attack.

The position of the second tartrate is compatible with the distance between 5′ and 3′ phosphates flanking a nucleotide in DNA. It is, therefore, possible to model the 5′-most nucleotide of a DNA substrate, with the obligatory 5′ phosphate occupying part of the tartrate_2 pocket, while the 3′ phosphate (the phosphodiester that is cleaved by the exonucleolytic activity of SNM1A and B) can be positioned at the Tartrate_1 site, interacting with the zinc and the activated water/hydroxy group. This provides a plausible explanation of the requirement for a 5′ phosphate, an essential requirement for the action of both SNM1A and SNM1B digestion.

A striking difference between SNM1A and SNM1B is that while both are similar in overall structure, they differ markedly in their charge distribution, with SNM1A having a very pronounced area of positive potential surrounding the active site. Our previous work demonstrated that SNM1A, in contrast to SNM1B, becomes far more processive on substrates over 50-nucleotides (regardless of whether digestion is initiated from a blunt end or single-stranded nick) (10), and we suggest that the patch of positive charge may reduce the dissociation of the DNA from the enzyme after each cleavage event, providing a likely explanation for this substantial difference between the apparent processivity of the two enzymes (Supplementary Figure S4). We also performed extensive mutational analysis of residues outside the zinc-binding region and identified distal sites in both SNM1A and SNM1B that are required for efficient digestion of DNA (especially long molecules), which having no effect on the kinetics of a single cleavage reaction. This remarkable observation may indicate that the DNA molecule is bound in a wide groove along the SNM1 proteins, reaching out (at least) to the distal site. The structure and size of the groove are compatible with binding dsDNA, and are probably compatible with distorted DNA structures such as interstrand crosslinks and other adducts. This may account for the unique ability of SNM1 proteins to digest through such DNA lesions (10,14).

## ACCESSION NUMBERS

Coordinates and structure factors have been deposited in the Protein Data Bank under accession codes 4B87, 5AHR and 5AHO.

## Supplementary Material

SUPPLEMENTARY DATA

## References

[1] Symington L.S., Gautier J. (2011). Double-strand break end resection and repair pathway choice. Annu. Rev. Genet..

[2] Jiricny J. (2013). Postreplicative mismatch repair. Cold Spring Harbor Perspect. Biol..

[3] Tsutakawa S.E., Lafrance-Vanasse J., Tainer J.A. (2014). The cutting edges in DNA repair, licensing, and fidelity: DNA and RNA repair nucleases sculpt DNA to measure twice, cut once. DNA Repair (Amst).

[4] Yan Y., Akhter S., Zhang X., Legerski R. (2010). The multifunctional SNM1 gene family: not just nucleases. Future Oncol. (London, England).

[5] Cattell E., Sengerova B., McHugh P.J. (2010). The SNM1/Pso2 family of ICL repair nucleases: from yeast to man. Environ. Mol. Mutagen..

[6] Callebaut I., Moshous D., Mornon J.P., de Villartay J.P. (2002). Metallo-beta-lactamase fold within nucleic acids processing enzymes: the beta-CASP family. Nucleic Acids Res..

[7] Tavtigian S.V., Simard J., Teng D.H., Abtin V., Baumgard M., Beck A., Camp N.J., Carillo A.R., Chen Y., Dayananth P. (2001). A candidate prostate cancer susceptibility gene at chromosome 17p. Nat. Genet..

[8] Mandel C.R., Kaneko S., Zhang H., Gebauer D., Vethantham V., Manley J.L., Tong L. (2006). Polyadenylation factor CPSF-73 is the pre-mRNA 3′-end-processing endonuclease. Nature.

[9] Dumont M., Frank D., Moisan A.M., Tranchant M., Soucy P., Breton R., Labrie F., Tavtigian S.V., Simard J. (2004). Structure of primate and rodent orthologs of the prostate cancer susceptibility gene ELAC2. Biochim. Biophys. Acta.

[10] Sengerova B., Allerston C.K., Abu M., Lee S.Y., Hartley J., Kiakos K., Schofield C.J., Hartley J.A., Gileadi O., McHugh P.J. (2012). Characterization of the human SNM1A and SNM1B/Apollo DNA repair exonucleases. J. Biol. Chem..

[11] Ma Y., Pannicke U., Schwarz K., Lieber M.R. (2002). Hairpin opening and overhang processing by an Artemis/DNA-dependent protein kinase complex in nonhomologous end joining and V(D)J recombination. Cell.

[12] Hazrati A., Ramis-Castelltort M., Sarkar S., Barber L.J., Schofield C.J., Hartley J.A., McHugh P.J. (2008). Human SNM1A suppresses the DNA repair defects of yeast pso2 mutants. DNA Repair (Amst.).

[13] Dronkert M.L., de Wit J., Boeve M., Vasconcelos M.L., van Steeg H., Tan T.L., Hoeijmakers J.H., Kanaar R. (2000). Disruption of mouse SNM1 causes increased sensitivity to the DNA interstrand cross-linking agent mitomycin C. Mol. Cell. Biol..

[14] Wang A.T., Sengerova B., Cattell E., Inagawa T., Hartley J.M., Kiakos K., Burgess-Brown N.A., Swift L.P., Enzlin J.H., Schofield C.J. (2011). Human SNM1A and XPF-ERCC1 collaborate to initiate DNA interstrand cross-link repair. Genes Dev..

[15] van Overbeek M., de Lange T. (2006). Apollo, an Artemis-related nuclease, interacts with TRF2 and protects human telomeres in S phase. Curr. Biol..

[16] Lenain C., Bauwens S., Amiard S., Brunori M., Giraud-Panis M.J., Gilson E. (2006). The Apollo 5′ exonuclease functions together with TRF2 to protect telomeres from DNA repair. Curr. Biol..

[17] Demuth I., Digweed M., Concannon P. (2004). Human SNM1B is required for normal cellular response to both DNA interstrand crosslink-inducing agents and ionizing radiation. Oncogene.

[18] Freibaum B.D., Counter C.M. (2006). hSnm1B is a novel telomere-associated protein. J. Biol. Chem..

[19] Moshous D., Callebaut I., de Chasseval R., Corneo B., Cavazzana-Calvo M., Le Deist F., Tezcan I., Sanal O., Bertrand Y., Philippe N. (2001). Artemis, a novel DNA double-strand break repair/V(D)J recombination protein, is mutated in human severe combined immune deficiency. Cell.

[20] Pannicke U., Ma Y., Hopfner K.P., Niewolik D., Lieber M.R., Schwarz K. (2004). Functional and biochemical dissection of the structure-specific nuclease ARTEMIS. EMBO J..

[21] Iyama T., Lee S.Y., Berquist B.R., Gileadi O., Bohr V.A., Seidman M.M., McHugh P.J., Wilson D.M. 3rd (2015). CSB interacts with SNM1A and promotes DNA interstrand crosslink processing. Nucleic Acids Res..

[22] Bae J.B., Mukhopadhyay S.S., Liu L., Zhang N., Tan J., Akhter S., Liu X., Shen X., Li L., Legerski R.J. (2008). Snm1B/Apollo mediates replication fork collapse and S Phase checkpoint activation in response to DNA interstrand cross-links. Oncogene.

[23] Mason J.M., Sekiguchi J.M. (2011). Snm1B/Apollo functions in the Fanconi anemia pathway in response to DNA interstrand crosslinks. Hum. Mol. Genet..

[24] Salewsky B., Schmiester M., Schindler D., Digweed M., Demuth I. (2012). The nuclease hSNM1B/Apollo is linked to the Fanconi anemia pathway via its interaction with FANCP/SLX4. Hum. Mol. Genet..

[25] Chen Y., Yang Y., van Overbeek M., Donigian J.R., Baciu P., de Lange T., Lei M. (2008). A shared docking motif in TRF1 and TRF2 used for differential recruitment of telomeric proteins. Science.

[26] Lam Y.C., Akhter S., Gu P., Ye J., Poulet A., Giraud-Panis M.J., Bailey S.M., Gilson E., Legerski R.J., Chang S. (2010). SNMIB/Apollo protects leading-strand telomeres against NHEJ-mediated repair. EMBO J.

[27] Touzot F., Callebaut I., Soulier J., Gaillard L., Azerrad C., Durandy A., Fischer A., de Villartay J.P., Revy P. (2010). Function of Apollo (SNM1B) at telomere highlighted by a splice variant identified in a patient with Hoyeraal-Hreidarsson syndrome. Proc. Natl. Acad. Sci. U.S.A..

[28] Wu P., van Overbeek M., Rooney S., de Lange T. (2010). Apollo contributes to G overhang maintenance and protects leading-end telomeres. Mol. Cell.

[29] Ye J., Lenain C., Bauwens S., Rizzo A., Saint-Leger A., Poulet A., Benarroch D., Magdinier F., Morere J., Amiard S. (2010). TRF2 and apollo cooperate with topoisomerase 2alpha to protect human telomeres from replicative damage. Cell.

[30] Savitsky P., Bray J., Cooper C.D., Marsden B.D., Mahajan P., Burgess-Brown N.A., Gileadi O. (2010). High-throughput production of human proteins for crystallization: the SGC experience. J. Struct. Biol..

[31] Totrov M., Abagyan R. (2001). Rapid boundary element solvation electrostatics calculations in folding simulations: successful folding of a 23-residue peptide. Biopolymers.

[32] Kiakos K., Hartley J.M., Hartley J.A. (2010). Measurement of DNA interstrand crosslinking in naked DNA using gel-based methods. Methods Mol. Biol..

[33] Dorleans A., Li de la Sierra-Gallay I., Piton J., Zig L., Gilet L., Putzer H., Condon C. (2011). Molecular basis for the recognition and cleavage of RNA by the bifunctional 5′-3′ exo/endoribonuclease RNase J. Structure.

[34] Newman J.A., Hewitt L., Rodrigues C., Solovyova A., Harwood C.R., Lewis R.J. (2011). Unusual, dual endo- and exonuclease activity in the degradosome explained by crystal structure analysis of RNase J1. Structure.

