# Supplementary material for: The structures of the SNM1A and SNM1B/Apollo nuclease domains reveal a potential basis for their distinct DNA processing activities
Source: Nucleic Acids Res. 2015 Nov 17;43(22):11047–60. doi: 10.1093/nar/gkv1256 (PMC4678830; doi:10.1093/nar/gkv1256)
Supplement: SUPPLEMENTARY DATA [file supp_43_22_11047__index.html]

The structures of the SNM1A and SNM1B/Apollo nuclease domains reveal a potential basis for their distinct DNA processing activities — The structures of the SNM1A and SNM1B/Apollo nuclease domains reveal a potential basis for their distinct DNA processing activities — SUPPLEMENTARY DATA 

# The structures of the SNM1A and SNM1B/Apollo nuclease domains reveal a potential basis for their distinct DNA processing activities

## SUPPLEMENTARY DATA

- SUPPLEMENTARY DATA
