# Supplementary material for: The structures of the SNM1A and SNM1B/Apollo nuclease domains reveal a potential basis for their distinct DNA processing activities
Source: Nucleic Acids Res. 2015 Nov 17;43(22):11047–60. doi: 10.1093/nar/gkv1256 (PMC4678830; doi:10.1093/nar/gkv1256)
Supplement: SUPPLEMENTARY DATA [file supp_gkv1256_nar-02515-h-2015-File009.pdf]

Table S1:

Name	Sequence
20-mer top strand	5’-P-ATAATTTGATCATCTATTAT-3’
21-mer bottom strand	5’-Biotin-TATAATAGATGATCAAATTAT-P-3’
50-mer top strand	5'-P-<br>TTATAAATAAAATATTAAAACCAATTTGATCATCTATTATATATTTATTA-<br>3′
20-mer fluor substrate	5'-P-A[FluoresceinT]AATTTGA[BHQ1-T]CATCTATTAT-3'
21-mer ICL substrate	5’-P-ATAATTTGATCATCTATTAT-3’<br>3’-TATTAAACTAGTAGATAATAT-P-5’
14-mer ICL substrate	5’-P-GATCATCTATTAT-3’<br>3’-TATTAAACTAGTAGATAATAT-P-5’

Supplementary Figure Legends

Supplementary Figure 1:

Stereo views of representative regions of the final refined 2Fo-1Fc electron density maps contoured at 1.5 σ . The protein is shown in the stick format with SNM1A crystal form B (5AHR) shown in blue in the upper panel, SNM1A crystal form A (4B87) shown in orange in the central panel and SNM1B (5AHO) shown in pink in the lower panel.

Supplementary Figure 2: Superposition of the crystal structures of SNM1A (PDB:4B87), SNM1B (PDB:5AHO), and CPSF73 (PDB:2I7T).

Supplementary Figure 3: Structure-based sequence alignment of CPSF73, SNM1A and SNM1B. Secondary structure elements of SNM1B are depicted as cylinders (α helices in red, 3.10 helices in pink) and arrows (β strands) under the aligned sequences. Residues marked in red over yellow shading are identical, residues shaded in cyan are similar. The β strands of the MBL domain are numbered as in Fig 1A. Above the aligned sequences, conserved motifs 1-5, A and B are marked, as is the CASP domain. Asterisks denote Zn-coordinating residues, triangles denote residues that contact the tartrate molecules in SNM1B. Residues K904 and K906 in the CASP domain of SNM1A are marked with daggers above the alignment.

Supplementary Figure 4: Models of DNA bound to SNM1A and SNM1B. These are the same models shown in Fig 3C and 2D, with the protein surface coloured by electrostatic potential.

Supplementary Figure 5: K_M's and k_{cat}'s of SNM1A and its mutants calculated using Prism software and fitted to a Michaelis-Menten curve using initial velocities of the enzymes obtained from real-time fluorescence assay described in Materials and Methods. 0.24 nM of enzyme and 10, 25, 50,100, 150, 200, 300, 400, 500, 750, 1000 nM of single-stranded 21-mer DNA used. Data is from a minimum of three repeats and error bars show the standard error of the mean.

Supplementary Figure 6: (A) Crystal structure of SNM1A with the mutated residues in green, the K904A-K906T mutation in red, the MBL domain in dark grey, the β-CASP domain in light grey and the zinc ions in cyan. **(B)** Time-course nuclease assay of SNM1A and its mutants (0.8 nM) 21-mer single-stranded DNA (100 nM).

Supplementary Figure 7: (A) Crystal structure of SNM1B with the mutated residues in green, the S183A mutation in red, the MBL domain in dark grey, the β-CASP domain in light grey and the zinc ions in cyan. **(B)** Time-course nuclease assay of SNM1B and its mutants (0.4 nM) 21-mer single-stranded DNA (100nM) quenched at the indicated times. **(C)** Time-course nuclease assay of SNM1B and SNM1B-S183A (0.4 nM) mutant using 3' radio-labelled (α-^{32}P-dATP) 51-mer single-stranded DNA (100 nM), quenched at the indicated times. **(D)** EMSA with 3' radio-labelled 51-mer single-stranded DNA with increasing concentrations of SNM1B and SNM1B-S183A mutant. Enzyme incubated with DNA (1 nM) for 5 minutes at 37°C before quenching on ice.

Supplementary Figure 8: K_M's and k_{cat}'s of SNM1B and its mutants calculated using Prism software and fitted to a Michaelis-Menten curve using initial velocities of the enzymes obtained from real-time fluorescence assay described in Materials and Methods. 0.4 nM of enzyme and 10, 25, 50,100, 150, 200, 300, 400, 500, 750, 1000 nM of single-stranded 21-mer DNA used. Data is from a minimum of three repeats and error bars show the standard error of the mean.

Supplementary Fig 1

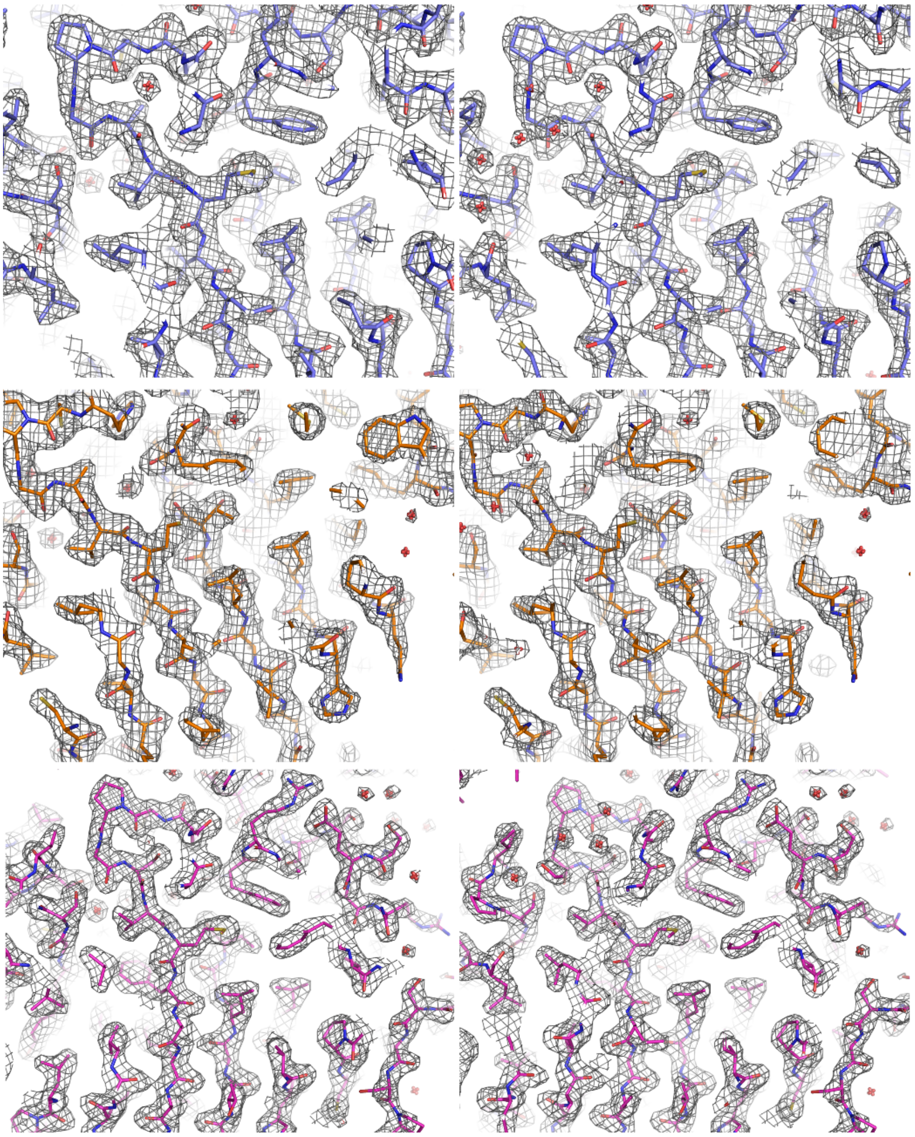

5AHR
(SNM1A)

4B87
(SNM1A)

5AHO
(SNM1B)

Supplementary Fig 2

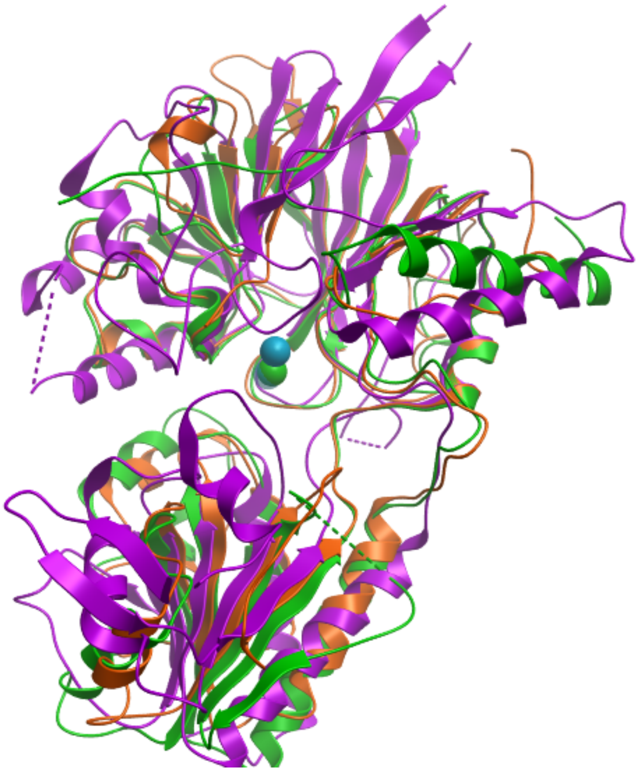

SNM1A: green
SNM1B: orange
CPSF73: purple

Supplementary Fig 3

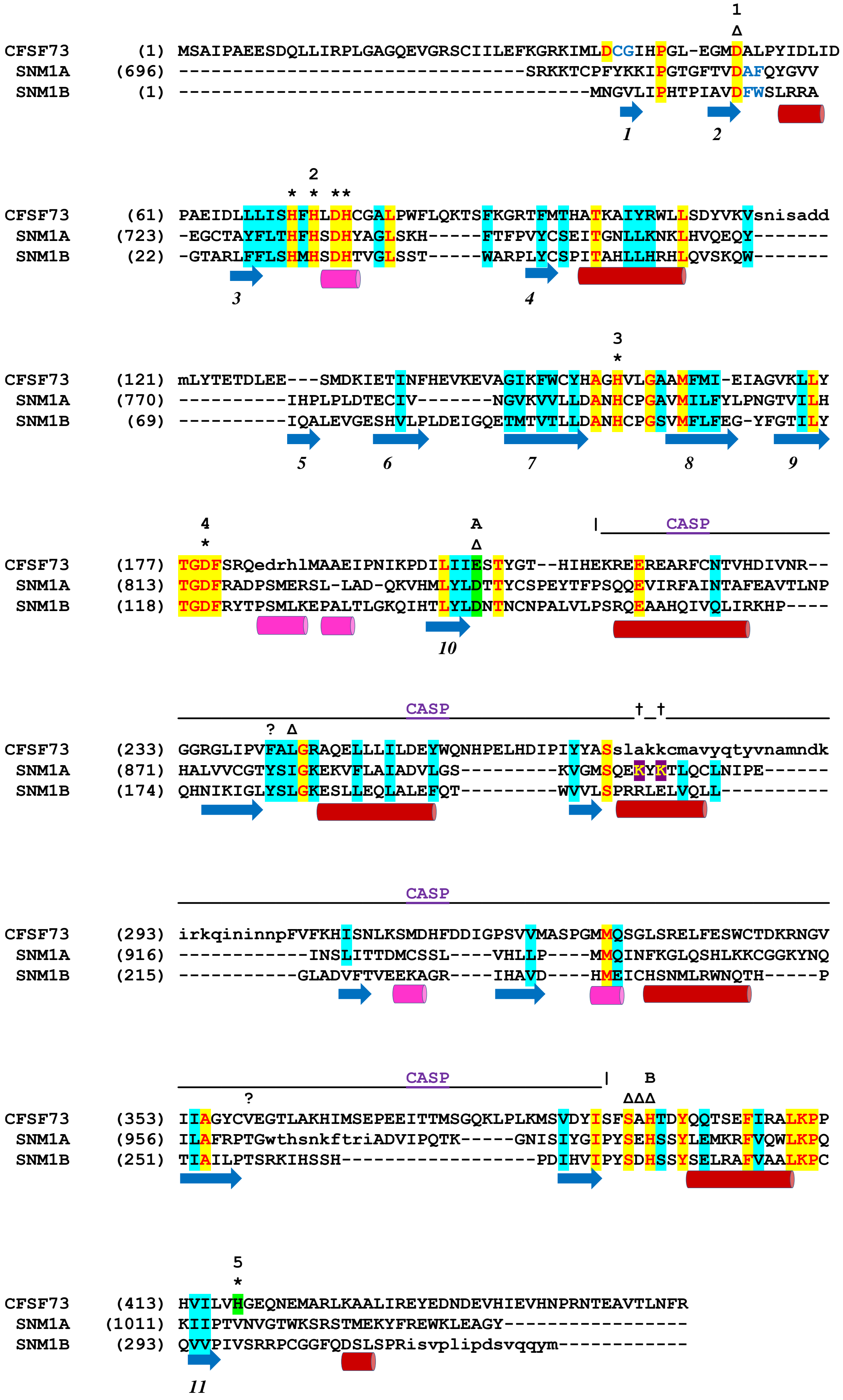

Supplementary Fig 4

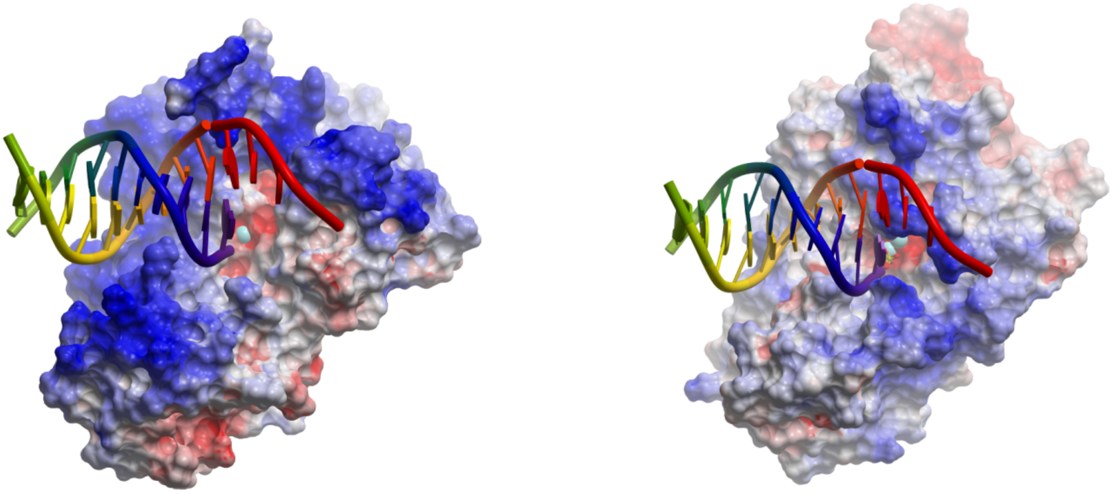

Supplementary Fig 5

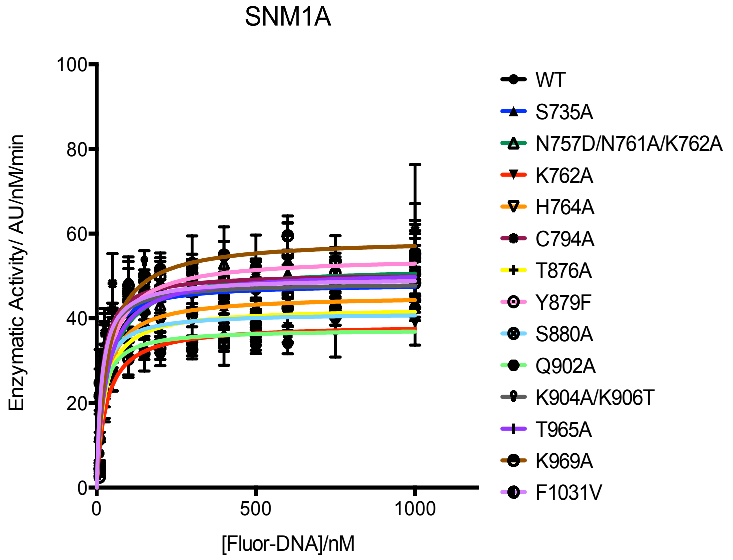

Mutant	K_M/nM	k_{cat}/AU/nM/min
Wild-type	28.4 ± 6.8	51.0 ± 2.0
S735A	17.1 ± 5.8	48.2 ± 2.3
N757D/N761A/K762A	35.2 ± 10.7	52.3 ± 2.8
K762A	30.0 ± 7.5	38.6 ± 1.6
H764A	25.1 ± 6.2	45.4 ± 1.8
C794A	15.5 ± 5.8	50.7 ± 2.6
T876A	24.8 ± 6.4	42.5 ± 1.7
Y879F	30.5 ± 8.1	54.5 ± 2.5
S880A	15.6 ± 6.0	41.3 ± 2.3
Q902A	16.5 ± 3.6	37.5 ± 1.2
K904A/K906T	14.7 ± 4.6	48.5 ± 2.0
T965A	30.5 ± 5.9	51.3 ± 1.7
K969A	32.3 ± 8.5	58.9 ± 2.8
F1031V	15.7 ± 4.3	49.5 ± 1.8

Supplementary Fig 6

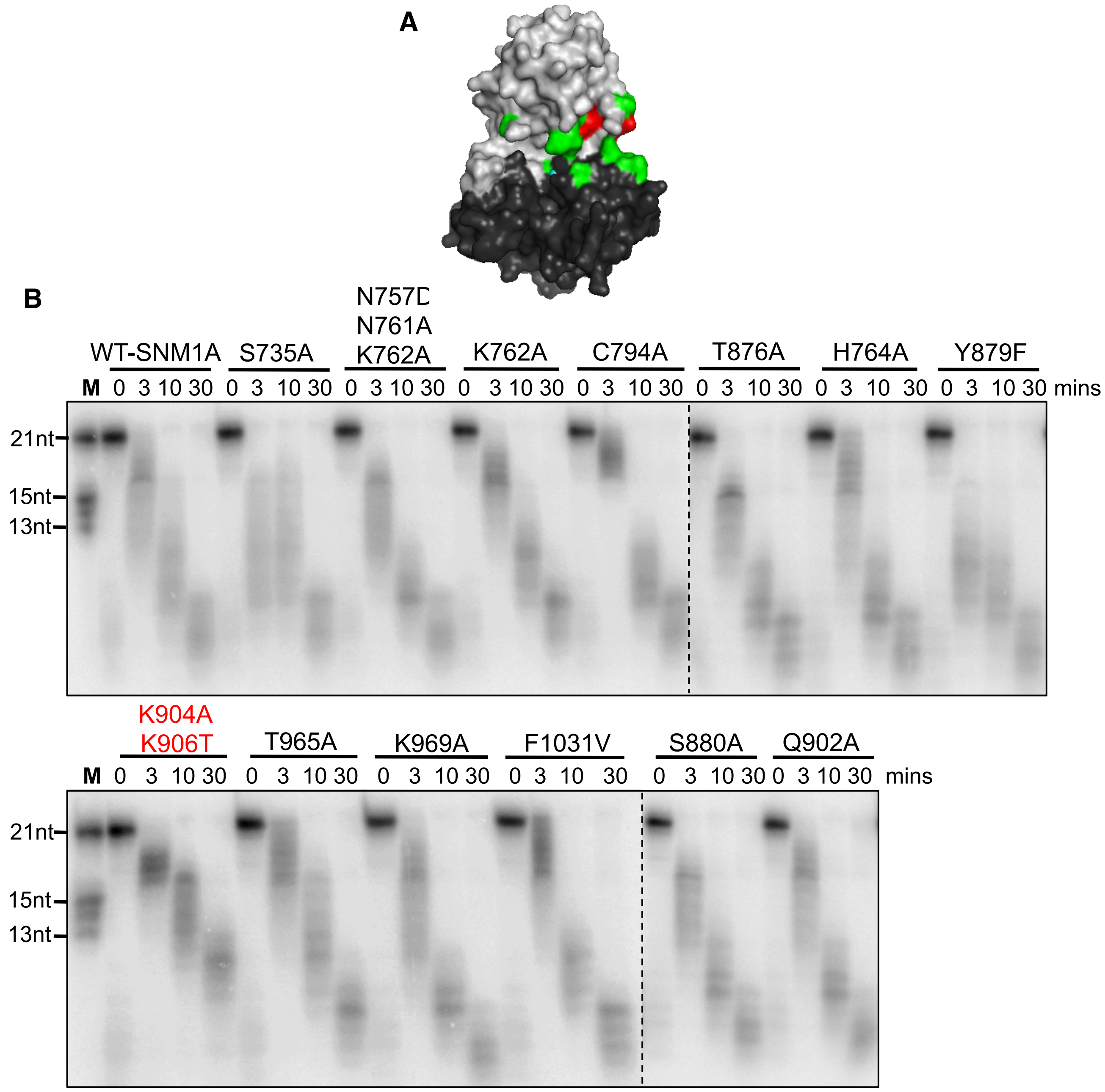

Supplementary Fig 7

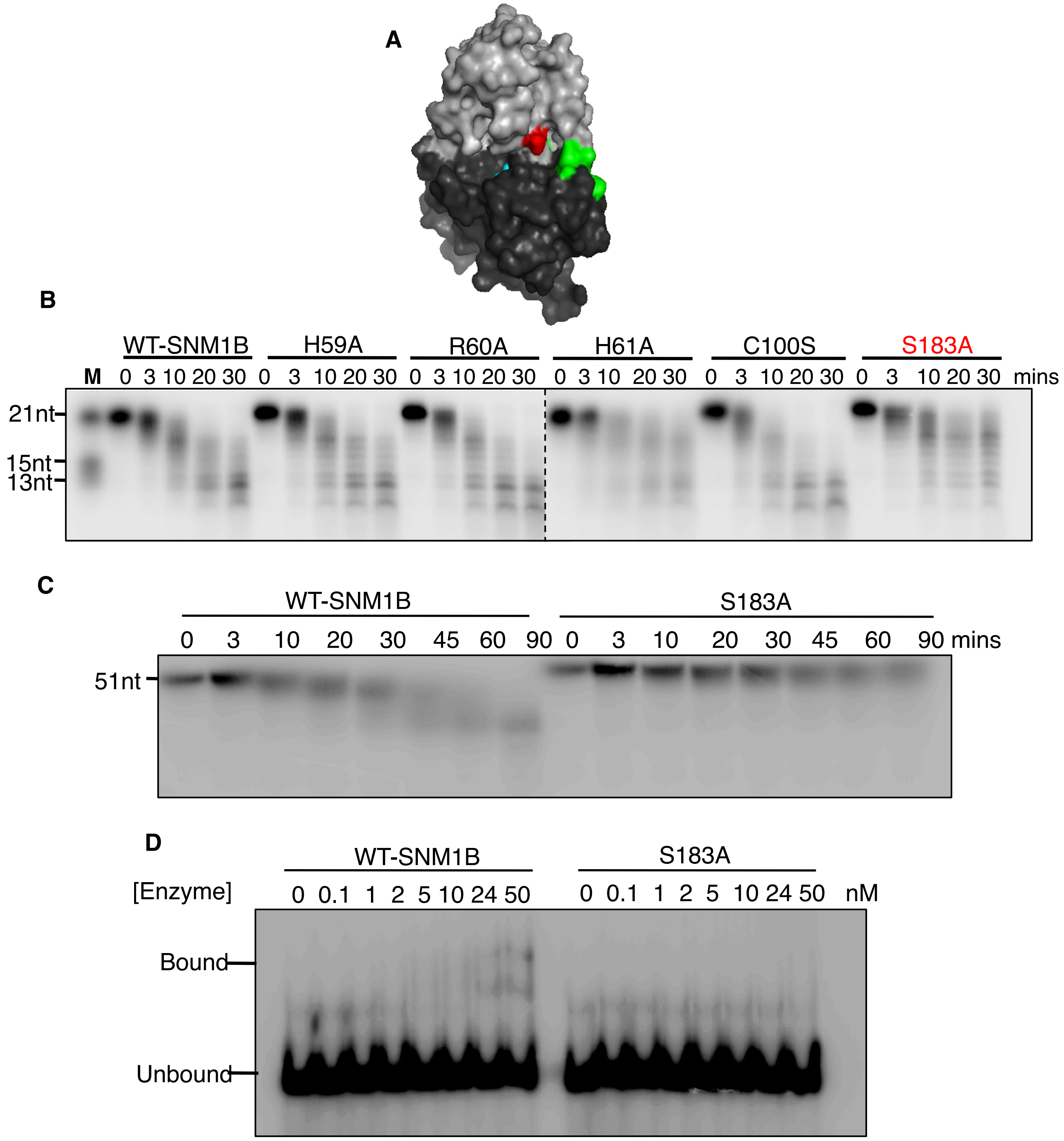

Supplementary Fig 8

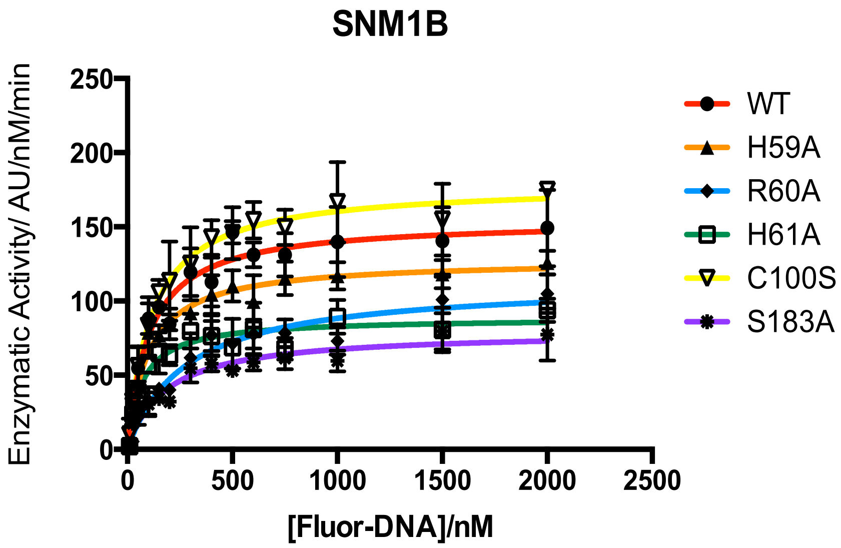

Mutant	K_M/nM	k_{cat}/AU/nM/min
Wild-type	103 ± 16	154 ± 5
H59A	97 ± 12	128 ± 3
R60A	278 ± 48	113 ± 6
H61A	68 ± 12	89 ± 3
C100S	113 ± 15	179 ± 6
S183A/S330F	181 ± 31	80 ± 4
